# The Use of Thoracic Segmental Spinal Anaesthesia for Thoracoscopic Diaphragmatic Hernia Repair in an Adult With Cardiac Compromise

**DOI:** 10.7759/cureus.56029

**Published:** 2024-03-12

**Authors:** Mahmoud R Manasra, Omar Q Heih, Rahaf F Adwan, Mohammed A Maraqa

**Affiliations:** 1 College of Medicine and Health Sciences, Palestine Polytechnic University, Hebron, PSE

**Keywords:** cabg, literature review, case report, morgagni hernia, hfref, segmental thoracic spinal anesthesia

## Abstract

Morgagni hernias are uncommon diaphragmatic defects and are commonly found incidentally as a congenital defect. Acquired Morgagni hernias have been documented in the pediatric population, making them extremely uncommon. Thoracic segmental spinal anesthesia (TSSA) may be used as a successful substitute for general anesthesia, especially in cardiovascularly compromised patients like our patient who had heart failure with reduced ejection fraction (HFrEF), and this is one of the very few documented cases of acquired Morgagni hernia laparoscopic repair surgery done by this anesthesia method.

An 80-year-old woman presented with a complaint of left lower chest and left upper quadrant pain. Acute coronary artery syndrome was ruled out. She underwent a coronary artery bypass graft in 2009, complicated two months later by mediastinitis, which is believed to be the cause of the acquired diaphragmatic defect in our patient. Chest and abdominal CT showed a large anterior Morgagni-type diaphragmatic hernia, in which the left hemithorax and anterior mediastinum were both occupied by a herniated transverse colon. Under regional anesthesia, which was done by injecting anesthesia in the spinal space between thoracic spinal vertebrae T8 and T9 and second injections in the epidural space at the level between thoracic epidural T9 and T10, which is neuraxial anesthesia. The repair of the diaphragmatic hernia was done by suturing the mesh into the proper position.

We report the first known case of laparoscopic repair of a Morgagni hernia in an adult patient with HFrEF and other comorbidities.

## Introduction

Morgagni hernia repair is commonly performed under general anesthesia (GA) [1]. While GA is becoming increasingly safe, there are still some dangers and problems; nevertheless, neuraxial anesthesia also has its risks and contraindications. The most frequent problems with general anesthesia are respiratory and cardiovascular in nature. Under GA, there is a chance of myocardial infarction [2], disruption of lung mechanics [3], and aggravation of preexisting comorbidities [4]. Thoracic segmental spinal anesthesia (TSSA) is a type of regional anesthesia that could serve as an effective alternative to general anesthesia in patients who have contraindications for GA. This neuraxial anesthesia technique is especially useful for individuals receiving general anesthesia who are deemed to be at high risk (e.g., old age). Improved patient safety, a shorter length of stay in the post-anesthesia care unit, and better postoperative pain control are some benefits of using thoracic spinal anesthesia for these routine operations [5]. For several surgical procedures, such as laparoscopic cholecystectomies [6], breast cancer lumpectomies [7], and abdominal cancer surgery [8], thoracic spinal anesthesia has been shown in the literature to be a safe and reliable technique [5]. As far as we know, we provide the first documented instance of laparoscopic repair of an acquired Morgagni hernia using TSSA in an 80-year-old woman with heart failure with reduced ejection fraction (HFrEF) and other comorbidities.

## Case presentation

**Figure 1:**
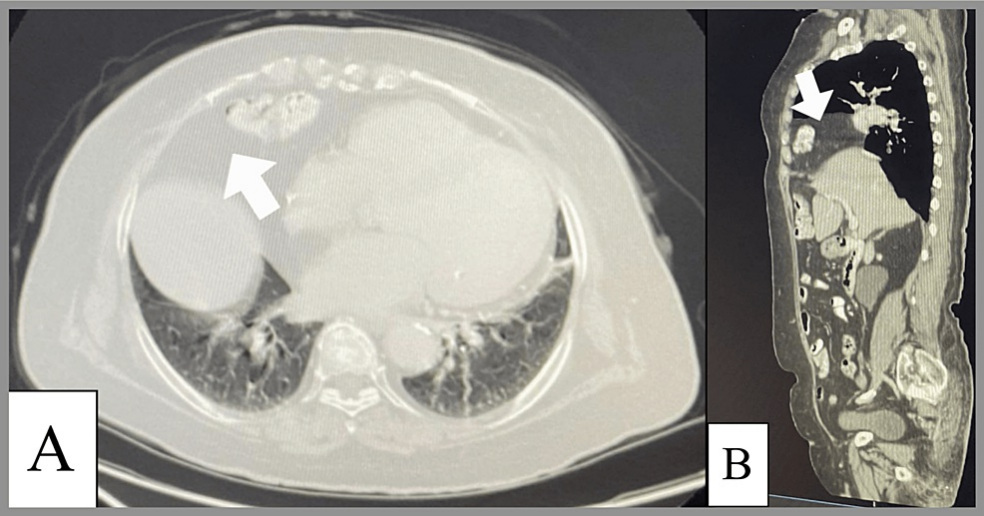
Cross-sectional plane abdomen computed tomography scan with contrast (A) and sagittal plane abdomen and pelvis CT scan IV contrast (B) shows a large defect at the anteromedial aspect of the right hemidiaphragm, measuring about 6x3 cm, through which the omental fat and part of the transverse colon are herniated into the right hemithorax, representing Morgagni hernia

The patient was an 80-year-old female in a wheelchair with a history of diabetes mellitus, congestive heart failure with a reduced HFrEF of 30%, hypertension, hyperlipidemia, and rheumatoid arthritis who presented with complaints of left lower chest and left upper quadrant pain. Over the past few weeks, the patient started to complain of left upper quadrant abdominal pain, which increased in severity five days before admission with acute onset, was progressive in coarse, not radiated, stepped in nature, exacerbated with effort, relieved with rest, had a severity of 10/10, and was associated with shortness of breath and dyspnea. On the exam, the patient was conscious and alert-oriented, with decreased air entry basally bilaterally, bilateral wheezes, and no crepitations. There was severe left-sided chest tenderness, soft abdomen, no audible heart murmur, no organomegaly, and no lower limb edema. She presented to a hospital, and acute coronary syndrome was ruled out based on clinical findings. In 2009, the patient had a coronary artery bypass graft performed via median sternotomy, which was complicated by mediastinitis two months later, necessitating the reopening of the same scar. Past surgical history also included renal surgery for stage horn stones and diseconomy. Four years later, the patient underwent six catheterizations with five stents. On presentation, the patient underwent chest and abdominal CT that showed a large anterior Morgagni-type diaphragmatic hernia with a herniated transverse colon occupying the anterior mediastinum as well as the left hemithorax (Figure 1). The plan was hospitalization for pain control and complete blood work: CBC, blood gases, lactate, liver function tests, and serum electrolytes. Based on the results, the patient needed surgical ICU management, thoracic surgery management, and general surgery management for possible urgent intervention in the form of a thoracotomy and/or laparotomy with the repair of the Morgagni hernia. Regional anesthesia was done by injecting the anesthesia into the spinal space between the thoracic spinal T8-T9 and the second injection in the epidural space at the level between thoracic epidural segments T9-T10, which is neuraxial anesthesia. The purpose was to decrease the dose of anesthesia medications, which led to a decrease in the side effects for the patient.

We used 1.5 cc of 0.5% isobaric bupivacaine with 5 mcg dexmedetomidine for TSSA. With the patient in a sitting position, TSSA was performed under due asepsis using a 25-gauge non-cutting (pencil point tip). The onset of the block was five minutes. For this surgery, the patient was not intubated. We kept everything ready for intubation in case of a failed block or any other reason for not proceeding under TSSA like patient discomfort or a patchy block. Drugs used to treat hypotension and bradycardia like ephedrine, atropine, and even phenylephrine were kept ready. An arterial line was secured for real-time monitoring of arterial blood pressure given the cardiac dysfunction. Throughout the procedure, the hemodynamics were stable and required no intervention, and the patient was comfortable and tolerated the entire procedure well.

**Figure 2:**
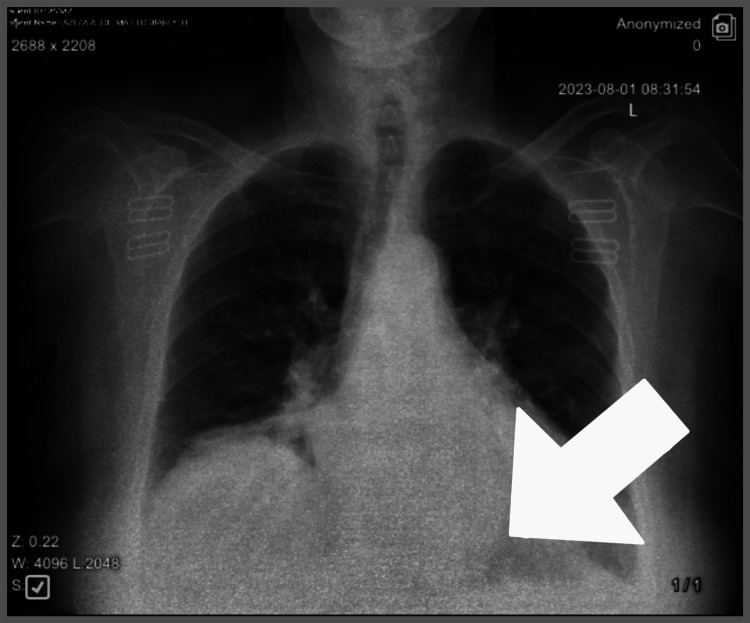
Chest X-ray after surgery showing the full resolution of the anterior diaphragmatic defect

The patient was placed in a supine position (reverse Trendelenburg position), and upon entering the abdomen with the laparoscope, we noted a large anterior diaphragmatic hernia containing the transverse colon and omentum and a defect opening in the diaphragm with the peritoneum bulging into the pleural cavity. The repair of the diaphragmatic hernia was done with suturing, and the mesh was appropriately positioned and then sutured into place. After the end of the procedure, a chest X-ray was done and showed full resolution of the anterior diaphragmatic defect (Figure 2).

## Discussion

Diaphragmatic hernias fall into one of two categories: acquired or congenital. Bochdalek hernias account for 80% of congenital diaphragmatic hernias while Morgagni hernias account for 2-6% [9-11]. Even more uncommon are acquired Morgagni hernias, which can be brought on by pregnancy, obesity, chronic constipation, persistent coughing, blunt thoracoabdominal trauma, or direct penetrating damage to the diaphragm that results in herniation [10,12]. Adult population symptoms can vary in how they present. According to a thorough literature review conducted by Horton and colleagues, the majority of patients arrive with pulmonary symptoms, such as dyspnea, coughing, and shortness of breath (36%), as well as chest and abdominal pain or pressure (37%) [10]. Only pediatric patients have been reported to develop Morgagni hernias after open cardiac surgery [10,13,14]. Our patients have no history of penetrating or blunt thoracoabdominal trauma and are without risk factors for the development of acquired Morgagni herniation; therefore, the likely cause is mediastinitis because the patient had reopened the sternotomy incision two months postoperatively after a complicated CABG procedure 14 years prior. As far as we are aware, this is the second known case of an adult diaphragmatic hernia of Morgagni type following heart surgery. The first case was published by Tamesis SA et al. in 2022 [15]. The most effective treatment for them is surgery. While laparoscopic surgery is preferred in elective settings and is linked to a shorter hospital stay, open surgical approaches are better in emergencies. One surgical principle that is thought to reduce the risk of a hernia recurrence is the excision of the hernia sac [16]. Mesh reinforcement or interposition can be the primary method used for diaphragmatic repair once the hernia's contents have been reduced and the defect has been identified. Thoman et al. recommend prosthetic repair for defects with a surface area greater than 20-30 cm^2^, so the decision to repair or use mesh largely depends on the size of the defect [17].

During Morgagni hernia repair surgeries, two types of anesthesia are used: general and regional (spinal or epidural). Given the relative advantages of GA over regional anesthesia, the requirement for adequate muscular relaxation for GA is the most widely used and sensible choice. However, spinal anesthesia is advised in patients who have cardiac compromise [18].

For GA, tracheal intubation, artificial breathing, and the use of neuromuscular blockers are all required. Numerous problems, such as atelectasis, lung infiltration, bronchospasm, ventilation-perfusion mismatch, and reduced respiratory muscle performance due to residual muscular relaxation, might result from these procedures. In addition to decreasing cardiac output, positive-pressure breathing also compromises the venous return to the heart. For abdominal procedures, regional anesthesia is adequate and reduces the possibility of these issues [19].

In fact, TSSA has advantages over general anesthesia as Elkany MH et al. published in breast cancer surgery [7]. TSSA is a type of regional anesthesia that may be a good substitute for general anesthesia and has shown effectiveness in many known published cases. This is particularly valid for patients undergoing general anesthesia who are thought to pose a high risk. According to a thorough review of the literature conducted by Harris and colleagues [4], the majority of complications associated with general anesthesia are cardiopulmonary in origin. These include heart failure, cardiac arrest, arrhythmias, thromboembolism, aspiration, bronchospasm, and other complications such as neurologic, renal, and others [4]. About 5% of these complications occur within the first 48 hours following surgery. Although the technique is relatively well-known, it is not frequently used in a wide range of surgical procedures. Nonetheless, studies have shown that it can help these patients maintain hemodynamic stability and lessen the negative effects of general anesthesia [5]. Anesthesiologists are hesitant to administer spinal anesthesia above the conus medullaris termination due to their fear of harming the spinal cord. Thoracic spinal anesthesia, however, has proven to be a safe and effective method for various procedures. When faced with significant medical issues, such as when a patient has severe lung disease or needs breast surgery, our multidisciplinary team opted to handle our case using this approach for the first time. We report the first case of laparoscopic repair of a Morgagni hernia in a patient with HFrEF and other comorbidities using the TSSA technique.

## Conclusions

Although thoracic segmental spinal anesthesia is not used routinely and has some limitations when used for abdominal surgeries, e.g., hypotension and risk of spinal cord injury, this case report demonstrates that TSSA may be a reasonable substitute for the use of conventional general anesthesia during laparoscopic repair of an acquired Morgagni hernia in a patient with cardiac compromise. However further studies are needed to determine other required parameters to perform TSSA for cardiac compromise patients. It is safe and more effective than GA in providing pain-free intervals after surgery, reducing the need for analgesics and opioids, and lowering the risk of postoperative cardiopulmonary complications. Although each patient is unique and there is a wide set of anesthetic options when managing conventional cases, the scenario described in this case report can help anesthesiologists expand their boundaries when managing complex cases.

## References

[1] Zhang M, Wang H, Liu D, Pan X, Wu W, Hu Z, Zhang H (2016). Non-intubated laparoscopic repair of giant Morgagni's hernia for a young man. J Thorac Dis.

[2] Devereaux PJ, Xavier D, Pogue J (2011). Characteristics and short-term prognosis of perioperative myocardial infarction in patients undergoing noncardiac surgery: a cohort study. Ann Intern Med.

[3] Smetana GW (2009). Postoperative pulmonary complications: an update on risk assessment and reduction. Cleve Clin J Med.

[4] Harris M, Chung F (2013). Complications of general anesthesia. Clin Plast Surg.

[5] Shatri G, Singh A (2023). Thoracic Segmental Spinal Anesthesia. https://www.ncbi.nlm.nih.gov/books/NBK572087/.

[6] van Zundert AA, Stultiens G, Jakimowicz JJ, Peek D, van der Ham WG, Korsten HH, Wildsmith JA (2007). Laparoscopic cholecystectomy under segmental thoracic spinal anaesthesia: a feasibility study. Br J Anaesth.

[7] Elakany MH, Abdelhamid SA (2013). Segmental thoracic spinal has advantages over general anesthesia for breast cancer surgery. Anesth Essays Res.

[8] Ellakany MH (2014). Thoracic spinal anesthesia is safe for patients undergoing abdominal cancer surgery. Anesth Essays Res.

[9] Carballo C, Dehmer J, Weiner T (2021). Acquired Morgagni diaphragmatic hernia and pericardial hernia after cardiac surgery. J Pediatr Surg Case Rep.

[10] Mohamed M, Al-Hillan A, Shah J, Zurkovsky E, Asif A, Hossain M (2020). Symptomatic congenital Morgagni hernia presenting as a chest pain: a case report. J Med Case Rep.

[11] Ağalar C, Atila K, Arslan NÇ, Derici ZS, Bora S (2019). Adult Morgagni hernia: a single center experience of 5 cases and review of literature. Turk J Surg.

[12] Thomas VP (2012). A rare case of Morgagni diaphragmatic hernia presenting in pregnancy. Indian J Surg.

[13] Bawazir OA, Mahomed A, Fayyad A, Bagaryn E, Bawazir A, Mandora R (2020). Laparoscopic-assisted repair of Morgagni hernia in children. Ann Pediatr Surg.

[14] Panda BR, Sumangala SG, Katewa A, Naik SK, Mishra J, Kumar RK (2010). Intrapericardial diaphragmatic hernia after arterial switch operation. Ann Thorac Surg.

[15] Tamesis SA, Ayazi S, Komatsu Y, Allen M, Jobe BA (2022). Acquired Morgagni hernia following coronary artery bypass graft (CABG) with successful robotic repair of hernia. Int J Surg Case Rep.

[16] Pfannschmidt J, Hoffmann H, Dienemann H (2004). Morgagni hernia in adults: results in 7 patients. Scand J Surg.

[17] Thoman DS, Hui T, Phillips EH (2002). Laparoscopic diaphragmatic hernia repair. Surg Endosc.

[18] Zahoor MU, Masroor R, Khurshid T, Azhar R, Amjad Yasin MM (2011). Thoracic epidural anaesthesia for open cholecystectomy. J Coll Physicians Surg Pak.

[19] Savas JF, Litwack R, Davis K, Miller TA (2004). Regional anesthesia as an alternative to general anesthesia for abdominal surgery in patients with severe pulmonary impairment. Am J Surg.

